# Efficacy of pretreatment with remimazolam on prevention of propofol-induced injection pain in patients undergoing gastroscopy

**DOI:** 10.1038/s41598-023-47151-3

**Published:** 2023-11-11

**Authors:** Ruimei Yuan, Jing Wen, Qingfei Xing, Lei Chao, Chunhai Hu, Jie Ren, Fanjun Meng

**Affiliations:** 1 Department of Anesthesiology, Jinan Central Hospital, Central Hospital Affiliated to Shandong First Medical University, Jinan, Shandong China; 2 Department of Urology, Jinan Central Hospital, Central Hospital Affiliated to Shandong First Medical University, No.105 JieFang Road, Jinan, 250013 Shandong China

**Keywords:** Drug discovery, Gastroenterology

## Abstract

To evaluate the efficacy of remimazolam pretreatment in preventing propofol-induced injection pain (PIP) in patients undergoing gastroscopy. One hundred and forty patients (ASA I–II, aged 18–65 years, BMI 18–28 kg/m^2^) who were to undergo gastroscopy were randomized into either a saline group (group S) or a remimazolam group (group R) (n = 70 for each) on a computer-generated random number basis. The patients in group S received normal saline (0.1 ml/kg) and those in group R were administered remimazolam (0.1 mg/kg) via intravenous infusion for 60 s. 30 s after the injection of normal saline or remimazolam, patients received intravenously propofol (0.5 ml/s) until loss of consciousness. A different anesthesiologist who was unaware of the pretreatment was responsible for maintaining the outcome. The primary endpoint of our study was the incidence of PIP, which was measured using a 4-point scale. Secondary endpoints include the intensity of PIP, vital signs, characteristics of surgery and recovery, and adverse events. The incidence of PIP was significantly lower in group R than in group S (13 *vs* 51%, *p* < 0.001), and a lower percentage of patients presented with moderate PIP (3 *vs* 20%, *p* < 0.001). Moreover, lower consumption of propofol, shorter recovery time, and greater patient satisfaction were observed in group R than in group S. Pretreatment with remimazolam can effectively reduce the incidence and intensity of PIP in gastroscopy and shorten the recovery time without severe adverse effects.

Clinical Trials Registration: Trial Registration: Chinese Clinical Trial Registry (identifier: ChiCTR2200063793). Registry time: 16/09/2022. Registry name: Efficacy of Pre-Treatment with Remimazolam on Prevention of Propofol-Induced Injection Pain in Patients Undergoing Gastroscopy. The date of patient enrollment began from 2022-9-17 to 2022-10-10. The link to the registration: https://www.chictr.org.cn/showproj.html?proj=176004.

## Introduction

Currently, gastroscopy is one of the most common outpatient procedures. With the development and improvement of diagnosis and treatment, an increasing number of patients are selecting procedural sedation for gastroscopy. Propofol has been widely used as an anesthetic because of its rapid onset and the short duration of its pharmacological action^1,2^. However, except for the hemodynamic instability caused by propofol, another concern for anesthesiologists is propofol-induced injected pain (PIP). As we know the incidence of PIP in adults is 28–90%^3^, which is considered to be the seventh undesirable problem in clinical practice of anesthesia after incision pain, nausea, vomiting, preoperative anxiety, intravenous catheterization discomfort and chills^4,5^. Many strategies have been applied to alleviate this pain, but the effects are various^6–12^. So there is a need for searching new drugs to decrease the incidence of PIP.

Remimazolam toluene sulfonate is a novel, water-soluble, ultra-short-acting anesthetic and sedative drug. As a benzodiazepine agonist targeting the γ-aminobutyric acid A (GABA_A_) receptor, it offers more rapid recovery and faster restoration of cognitive function than midazolam. It is in contrast to all the other benzodiazepines because of its high clearance, small volume of distribution, and can be ester hydrolysed by carboxylesterase-1 to an inactive carboxylic acid metabolite. Additionally, it allows for prolonged infusions without the obvious accumulation or pharmacological action of metabolites^13^. Furthermore, the effects of remimazolam can be fully reversed by flumazenil^14^. Recent studies have reported that remimazolam is suitable for short operations, such as procedure sedation for gastroscopy, and its efficacy is non-inferior to propofol^15^. Both propofol and remimazolam play a role in inhibiting neuronal activity by activating the central GABA_A_ receptor^16,17^. A combination of the two drugs might act synergistically to enhance sedation during gastroscopy and reduce the incidence of hemodynamic side effects. In our early clinical application, it was found that the incidence of PIP was lower when the two drugs were combined than when propofol was used alone. This study, therefore, aimed to explore the inhibitory effect of remimazolam pretreatment on PIP in gastroscopy, thus providing a reference for clinical practice.

## Methods

This is a prospective, single-center, randomized, double-blinded, and placebo-controlled clinical trial registered in the Chinese Clinical Trial Registry (ChiCTR2200063793). Our study obtained approval from the Ethics Committee of Jinan Central Hospital (2022-107-02) and all methods were performed in accordance with the relevant guidelines and regulations. The total sample size was one hundred and forty patients (seventy patients per each group). Patients were randomly scheduled by using Epicalc 2000 soft and divided into two groups in a 1:1 group allocation to receive either normal saline (Group S) or remimazolam (Group R). We included patients (ASA I–II, aged 18–65 years, BMI 18–28 kg/m^2^) who were to undergo procedural sedation during gastroscopy regardless of gender. We excluded patients with recent upper respiratory tract infection and asthma, benzodiazepines and propofol susceptibility, nervous system or cardiovascular diseases, suspected abuse of narcotic analgesics or sedatives, difficult airway, pregnant and lactation and men with family planning in recent 3 months. All selected patients were informed about the purpose of the trial and written consent was obtained.

Patients were fasted for 8 h and only clear liquids were allowed up to 2 h before the induction of anesthesia. After the patient entered the examination room, peripheral venous access was established in the upper extremity. The patient was placed in the left decubitus position, and oxygen was received through the nasal catheter with 4 l/min. Vital signs such as noninvasive blood pressure (BP), electrocardiogram, heart rate (HR), and peripheral capillary oxygen saturation (SpO_2_) were monitored. Patients were divided into two groups: Group S (n = 70), which was injected with normal saline 0.1 ml/kg in advance within 60 s; In group R (n = 70), remimazolam 0.1 mg/kg was injected intravenously also within 60 s. 30 s after the injection of normal saline or remimazolam, patients were received intravenously propofol at the rate of 0.5 ml/s until loss of consciousness. Drugs used in the study: Propofol injection (20 ml: 200 mg, Sichuan Guorui Pharmaceutical Co., Ltd., batch number: 2201233); Remimazolam, toluene sulfonate (36 mg, Jiangsu Heng Rui Pharmaceutical Co., Ltd., batch number: 200205AK); 0.9% sodium chloride injection (100 ml: 0.9 g, China Otsuka Pharmaceutical Co., Ltd., batch number: 5J80F2). The pre-treating drugs were prepared in a 10 ml syringe with either 10 ml of normal saline or remimazolam diluted with normal saline (1 mg/ml) to 10 ml. Sealed envelopes were selected for concealment of the study group allocation until the drug was prepared. The assistor who prepared all drugs did not participate in anesthesia induction. Both patients and investigators were blinded to the randomized grouping allocation and the drugs. All prepared drugs were stored at room temperature and used in 10 min.

The Ambesh four-point pain score method^18^ was used by investigators who were blinded to the group location to evaluate the severity of PIP. The patient was repeatedly asked about the intensity of PIP every 5 s during the induced anesthesia of propofol: grade 0, no pain (the patient complained of no pain at the injection site after repeated questioning); grade 1, mild pain (the patient complained of pain through the doctor's initiative, but no physical movement); grade 2, moderate pain (voluntarily complaining of pain at the injection site to the doctor, or self-reported pain accompanied by physical activity when the anesthetist asked); grade 3, severe pain (facial pain, painful expression, accompanied by strong vocal response, arms retracted, or tears).

The primary endpoint variable in this study was the incidence of PIP. Secondary endpoints included the intensity of PIP, patient satisfaction of anesthesia, vital signs, characteristics of surgery and recovery, and adverse events, including hypotension (≤ 80% of basic blood pressure), hypoxemia (SpO_2_ < 90%), chin lifting (SpO_2_ < 90%), bradycardia (< 50 beats/min), physical movement, cough, nausea and vomiting. BP, HR, SpO_2_, and other vital signs were recorded at the following time points: before the pre-treatment with normal saline or remimazolam (T0), immediately after insertion of the gastroscope (T1), withdrawal of the gastroscope (T2), and the patient responds to the call for the first time (T3). The patient satisfaction of anesthesia was measured by a questionnaire^19^ about satisfaction (mild dizziness or none, mild nausea, vomiting or no) or dissatisfaction (dizziness, nausea, vomiting) by an assistor who did not participate in anesthesia induction. The questionnaire was collected before the patient leaving the Post-Anesthesia Care Unit. The definition of characteristics of surgery and recovery: the anesthesia time was defined as the time from the pre-treating drugs administrated to the end of gastroscopy; the surgery time was defined as the time from T1 to T2; the recovery time was defined as the time from the end of gastroscopy to T3.

The incidence of PIP in the previous studies was various from 28 to 90%, and our preliminary study found that about 50% in our department. We hypothesized a 50% reduction in the incidence of PIP based on an alpha of 0.05 and a power of 80%. 57 patients were included in each group to detect a significant difference under these assumptions. Considering 20% potential loss to follow-up, 70 patients were needed in each group.

All statistical analyses were analyzed with IBM SPSS 26.0 statistical software. All data are expressed as number (%) or mean ± SD. Continuous data of patients between the two groups were analyzed by independent sample *t*-test. Categorical data were compared by x^2^ test or Fisher’s test. *p*-values or corrected *p*-value of 0.05 were indicated as statistically significant.

## Results

**Figure 1:**
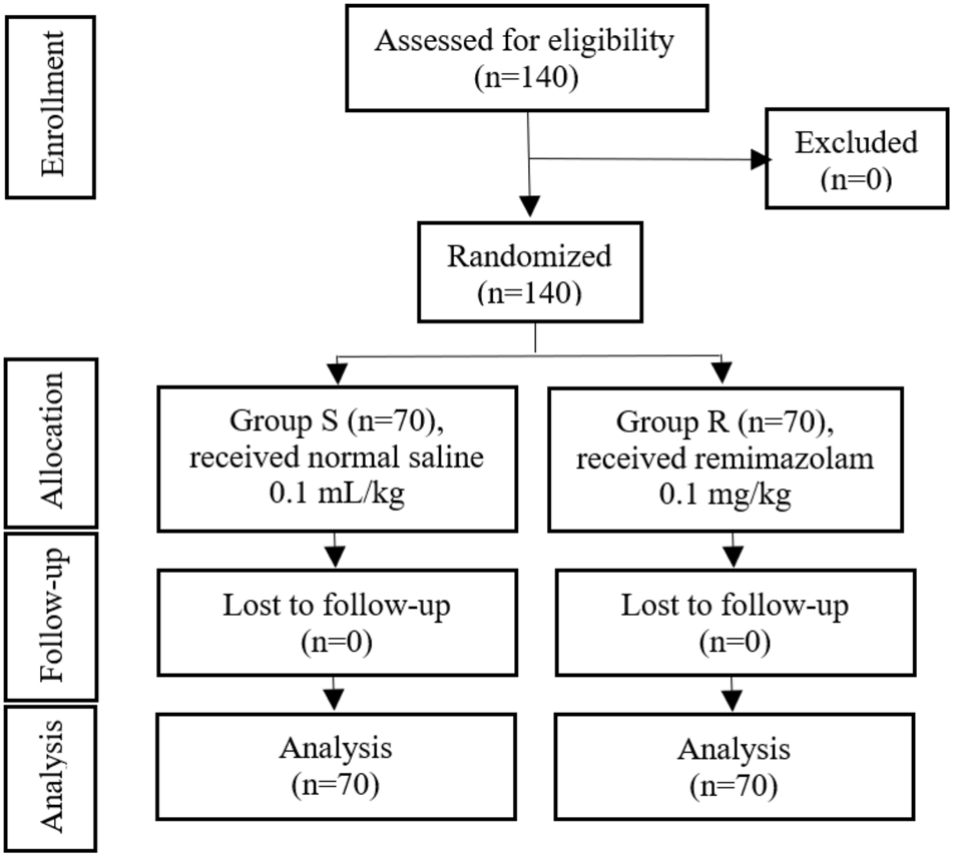
CONSORT flow of clinical procedures. S, normal saline; R, remimazolam.

In this clinical trial, data of all 140 patients were evaluable (Fig. 1; from 2022-9-17 to 2022-10-10). No significant differences between the two groups in demographics age, height, body weight, BMI, ASA score, rate, or gender (Table 1).

**Table 1:** Demographic data of patients (n = 70 in each group).

Parameter	Group S	Group R	*p* value
Age (years)	51.47 ± 10.80	47.17 ± 12.02	0.151
Height (cm)	164.6 ± 7.47	168.3 ± 9.10	0.088
Body weight (kg)	65.20 ± 10.16	70.13 ± 13.60	0.106
BMI (kg/m^2^)	24.93 ± 2.36	24.59 ± 3.29	0.372
ASA score(I/II)	29/41	34/36	0.396
Sex (M/F)	35/35	32/38	0.612

Notes: Data are displayed as means ± SD or number of cases. No statistically significant differences were noted between the two groups.

S, Normal saline; R, remimazolam.

**Figure 2:**
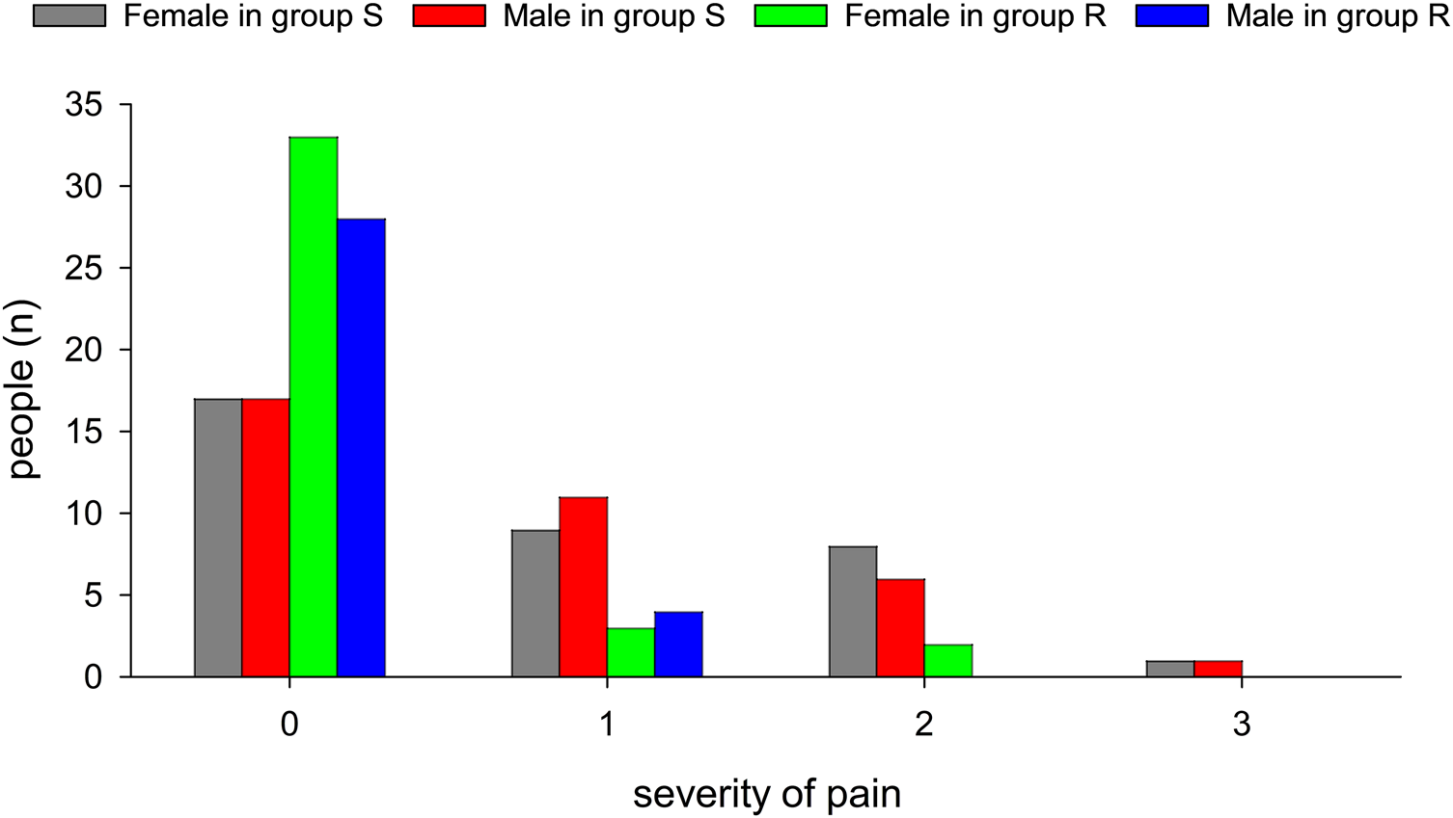
The difference of sex on the severity of pain between the two groups. Notes: Data are displayed as the number of cases. Severity of pain: 0, no pain; 1, mild pain; 2, moderate pain; 3, severe pain. S, normal saline; R, remimazolam.

The incidence and intensity of PIP during propofol injection and patient satisfaction of the gastroscopy in the two groups are shown in Table 2. The incidence of PIP was significantly lower in group R (13%) than that in group S (51%) (*p* < 0.001). Also, a lower percentage of patients presented with mild pain (10% *VS* 29%, *p* < 0.01), and presented with moderate pain (3% *VS* 20%, *p* < 0.001) in group R than in group S. There was also a significant difference between the two groups in the percentage of patients presented with severe pain, 0 cases and 2 cases were found in group R and group S, respectively. We also showed that patients receiving remimazolam in group R (83%) had greater patient satisfaction than those in group S (66%). No significantly difference was found in the role of sex on the severity of pain between the two groups in our study (Fig. 2).

**Table 2:** Incidence of propofol induced injection pain and patient satisfaction (n = 70 in each group).

Group	S	R
Patients with pain [No. (%)]	36 (51%)	9 (13%)*
Severity of pain [No. (%)]		
0	34 (49%)	61 (87%)*
1	20 (29%)	7 (10%)**
2	14 (20%)	2 (3%)*
3a	2 (3%)	0
Patient satisfaction [No. (%)]	46 (66%)	58 (83%)^#^

Notes: Data are displayed as the number of cases. Chi-square test were used to analyze the incidence of PIP. *p < 0.001, compared with group S, **P < 0.01, compared with group S, ^#^*p* < 0.05, compared with group S.

S, Normal saline; R, remimazolam.

The incidence of adverse events between the two groups is shown in Table 3. The percentage of patients with hypoxemia in group R (16%) was lower than that in group S (23%). No significant differences in the proportion of patients developing hypotension and bradycardia were observed between the two groups. Also, there were no significant differences between the two groups in the proportion of patients with cough, dizziness, nausea, and vomiting.

**Table 3:** Incidence of adverse events between two groups (n = 70 in each group).

Group	S	R
Hypoxemia (SpO_2_ < 90%)	16	11
Chin lift (SpO_2_ < 90%)	16	11
Hypotension	4	3
Bradycardia (< 50 beats/min)	0	0
Cough	8	6
Dizziness	7	4
Nausea and vomiting	3	3

Notes: Data are displayed as the number of cases. Chi-square test were used to analyze the incidence of an adverse events.

S, Normal saline; R, remimazolam.

The characteristics of anesthesia, surgery, and recovery time are shown in Table 4. The consumption of propofol in group R (117.4 ± 21.02 mg) was lower than that in group S (151.6 ± 28.57 mg) (*p* < 0.001). No significant difference was observed in the length of anesthesia and surgery between the two groups. The recovery time was shorter in group R (4.39 ± 3.28 min) than that in group S (6.12 ± 2.36 mg) (*p* < 0.001).

**Table 4:** Characteristics of anesthesia and surgery (n = 70 in each group).

Parameter	Group S	Group R	*p* value
Propofol dose (mg)	151.6 ± 28.57	117.4 ± 21.02*	0.000
Anesthesia time (min)	7.23 ± 2.86	7.95 ± 2.73	0.091
Surgery time (min)	5.15 ± 2.02	5.22 ± 1.83	0.082
Recovery time (min)	6.12 ± 2.36	4.39 ± 3.28*	0.000

Notes: Data are all displayed as means ± SD. Independent sample *t*-test was used to analyze all characteristics. *p < 0.001, compared with group S.

S, Normal saline; R, remimazolam.

**Figure 3:**
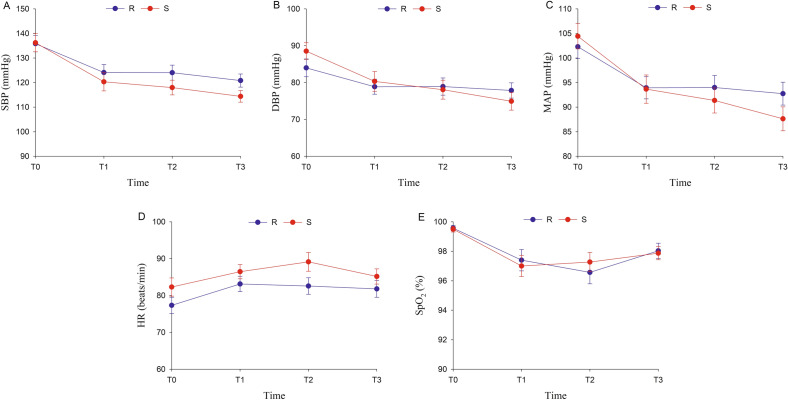
Changes of vital signs (**A**–**E**). Notes: Data are displayed as means ± SD. Time points: T0, at the time before the pre-treatment with normal saline or remimazolam; T1, immediately after insertion of the gastroscope; T2, withdrawal of the gastroscope; T3, the patient responds to the call for the first time. S, normal saline; R, remimazolam; SBP, systolic blood pressure; DBP, diastolic blood pressure; MAP, mean artery pressure; HR, heart rate; SpO_2_, peripheral capillary oxygen saturation.

There were no significant differences in systolic blood pressure (SBP), diastolic blood pressure (DBP), mean artery pressure (MAP), HR, and SpO_2_ at any time point between the two groups (Fig. 3).

## Discussion

We designed a prospective, single-center, randomized, double-blinded, and placebo-controlled to explore the efficacy of remimazolam prior to anesthesia induction with propofol to attenuate injection pain in patients under procedural sedation. According to the findings, it was evident that remimazolam pre-administration plus propofol had a favorable effect in alleviating propofol injection pain compared to normal saline. In Group R, patients reported significantly lower incidence and intensity rates of PIP, less propofol was necessary, the recovery time was shorter, and without severe adverse effects.

During the clinical application of propofol, except for hypotension and respiratory depression, the common adverse reaction is mainly the local irritating pain However, the definite patho-physiological mechanism of PIP remains unclear. Many factors have been described to be associated with PIP, such as the concentration of free propofol in the aqueous solution, type of preparation, oil and solvent, injection technology, blood buffering, pH value of propofol injection, filtration treatment, age, sex, and so on^5^. Additionally, numerous drugs have been shown to reduce the incidence of PIP. For example, Xing et al.^6^ found that a dosage of 40 mg lidocaine was an appropriate dosage to alleviate PIP within the same vein (around local anesthesia), while lidocaine 1.5 mg/kg injected through the contralateral arm vein also can relieve PIP in the other arm vein (around the central analgesia). Akbari et al.^20^ found that the incidence of PIP was 36%, 16%, and 4%, respectively, when 30 mg/kg magnesium sulfate, 0.5 mg/kg thiopental sodium and 0.5 mg/kg ketamine were injected through the ipsilateral dorsal vein 1 min before propofol injection. Similarly, dexmedetomidine^8^, opioids such as remifentanil^11,21^, dezocine^9^, 5-HT3 receptor antagonist^7^, non-steroidal anti-inflammatory drugs^22^, ephedrine^23^, esmolol^10^ exerts a certain amount of inhibition on PIP without effecting complete elimination when using only one intervention. As we know, PIP is immediate as well as delayed after 10–20 s, the immediate pain is because of the irritation of vein endothelium and delayed pain is because of the release of mediators such as kininogen from kinin cascade^5^. Though in this study, the consumption of propofol in group R (117.4 ± 21.02 mg) was lower than that in group S (151.6 ± 28.57 mg), while the incidence and intensity of PIP would not be reduced due to the lower consumption of propofol^5^. We believe that in the first 20 s of the propofol injection at a rate of 0.5 ml/s in both groups, may present similar blood concentration that contribute to PIP. In our study, the incidence of PIP after pretreatment with remimazolam is 13%, which is similar to the incidence of PIP after pretreatment with 0.5 mg/kg ketamine in Akbari et al.^20^. An increasing number of patients who undergo gastroscopy are elderly, and commonly have cardiac or respiratory comorbidities. Currently, two or more sedatives or anesthetic drugs are used for procedural sedation during gastroscopy in order to reduce the hemodynamic side effects. When remimazolam is combined with propofol in gastroscopy, it can play a synergistic role in sedation through rapid anesthetic effects and awakening and producing less of a suppressive effect on breathing, with lead to shorter recovery and hospital departure times, thereby providing to be safer and more effective^24^.

Remimazolam is a recently developed ultra-short-acting benzodiazepine agonist that has an organ-independent metabolism, similar to remifentanil, and acts on GABA receptors similar to midazolam^25^. As an ideal sedative, it offers more rapid recovery and earlier restoration of cognitive function, minor influence on liver and kidney function, hemodynamics, and rapid reversal by flumazenil. Guan et al.^26^ first indicate that pretreatment with remimazolam reduced the incidence and intensity of PIP in abortion or curettage patients, which was equivalent to that of lidocaine without severe adverse effects. The patients enrolled in their study with average age from 29.0 years (Remimazolam group) to 32.01 years (Lidocaine group). In the present study, both sexes were enrolled, and the average age was 47.17 years in group R and 51.47 years in group S. We found that sex, as a factor, was related to PIP, while no significant difference in PIP was found between the two groups. Age was another actor that contributed to the different incidence of PIP, 76% of patients with PIP according to Guan et al.^26^, as opposed to the 51% obtained in our study.

The possible mechanisms of reducing PIP with remimazolam may be: (1) by means of enhancing the synaptic inhibitory effects of GABAergic neurotransmission^26^. GABA_A_ receptors assemble from five protein subunits. Pretreatment with remimazolam acts on the GABA_A_ receptor, harboring α1 and β2 subunits^27^, to mediate sedative actions and on α2 subunits to mediate antihyperalgesic actions, which may contribute to preventing the occurrence of PIP. (2) Some studies have found remimazolam can reduce the release of pro-inflammatory cytokines^28,29^ and may reduce the incidence of PIP. However, the mechanism by which remimazolam reduces the incidence of PIP requires further investigation.

## Conclusions

To sum up, our study indicates that pretreatment with 0.1 mg/kg remimazolam can effectively reduce the incidence and intensity of PIP in gastroscopy. Moreover, pretreatment with 0.1 mg/kg remimazolam can also reduce propofol consumption and recovery time, without severe adverse effects.

## Data Availability

All data generated or analyzed during this study are included in this published article.
